# Supplementary material for: End Sequence Analysis Toolkit (ESAT) expands the extractable information from single-cell RNA-seq data
Source: Genome Res. 2016 Oct;26(10):1397–410. doi: 10.1101/gr.207902.116 (PMC5052061; doi:10.1101/gr.207902.116)
Supplement: Supplemental Material [file supp_gr.207902.116_Supp_Materials.tar.gz › ESAT-esat_v0.1/doc/html/namespacebroad_1_1pda_1_1seq_1_1segmentation.html]

ESAT: Package broad.pda.seq.segmentation


ESAT


- Main Page
- Related Pages
- Packages
- Classes
- Files

- Packages

All Classes Namespaces Files Functions Variables Enumerator Pages

- broad
- pda
- seq
- segmentation

Packages |
Classes

Package broad.pda.seq.segmentation

|  |  |
| --- | --- |
| Packages | |
| package | utils |
|  | |

|  |  |
| --- | --- |
| Classes | |
| interface | AlignmentDataModel |
|  | |
| class | AlignmentDataModelStats |
|  | |
| class | ContinuousDataAlignmentModel |
|  | |
| class | DefineSourceAndSinkFromPairedEnds |
|  | |
| class | GeneCounts |
|  | |
| class | GenericAlignmentDataModel |
|  | |
| class | GeneScore |
|  | |
| class | GenomeWithGaps |
|  | |
| class | GenomeWithGaps2 |
|  | |
| class | GlobalSpliceFilter |
|  | |
| class | PrecomputeDataAlignmentStats |
|  | |
| interface | ReadCountNormalizer |
|  | |
| interface | ReadFilter< T > |
|  | |
| class | WrappedIGVIterator |
|  | |


---

Generated on Fri Aug 15 2014 15:11:03 for ESAT by  

 1.8.7
